# Impact of backpack load during walking: an EMG and biomechanical analysis

**DOI:** 10.1007/s11517-024-03280-z

**Published:** 2025-01-11

**Authors:** Fırat Matur, Fatma Alnamroush, Bora Büyüksaraç

**Affiliations:** Biomedical Engineering, Bahçeşehir University, Çırağan Caddesi Osmanpaşa Mektebi Sokak No: 4-6 Beşiktaş, İstanbul, 34353 Turkey

**Keywords:** Backpack, Spinal joints, Back muscles, Muscle activation, Range of motion

## Abstract

**Abstract:**

This study aims to understand the impact of backpack carriage, a regular activity for many, on back muscles and joint mobility during walking so that clinicians can develop strategies or products to ensure individuals’ safety and well-being. Surface electromyography (EMG) and XSENS Awinda motion capture systems were used to analyze the effects of carrying a backpack (12% of body weight) on erector spinae and multifidus muscles, as well as spinal, hip, knee, and ankle joints. Subjects walked at 4 km/h on flat and inclined surfaces. Paired *t*-tests compared backpack loads to baseline measurements. Carrying a backpack reduced activation levels in erector spinae and multifidus muscles and restricted spinal joint range of motion (axial and lateral bending, $\varvec{p<0.05}$). Hip joint rotation increased ($\varvec{p<0.05}$). Moderate to strong correlations were observed between muscle activity and spinal joint ROM, notably with left erector spinae and L5-S1 lateral bending ($\varvec{r=0.723}, \varvec{p<0.001}$). Backpack carriage decreases back muscle activation and alters the joint range of motion. Asymmetric correlations show that the subjects adapt muscle activity and gait patterns asymmetrically to manage external loads.

**Graphical abstract:**

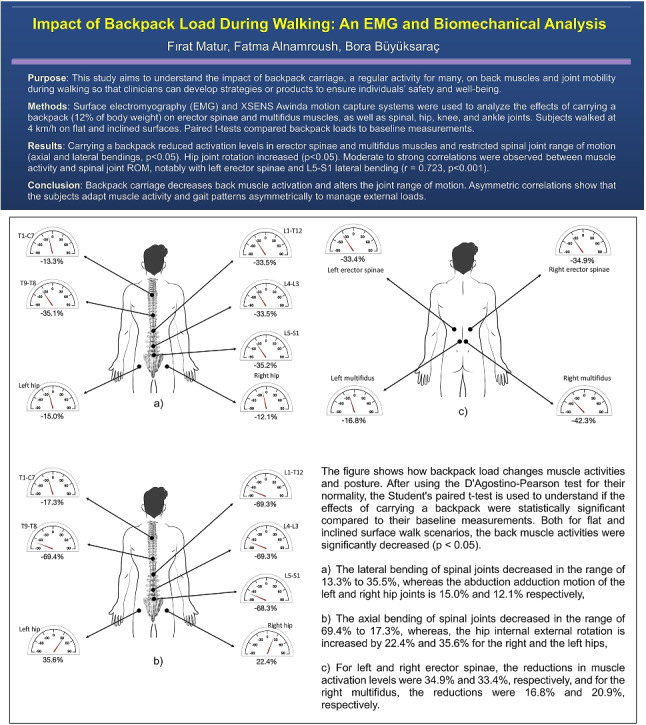

## Introduction

The muscles surrounding the spinal column in the back and the core play a key role in supporting the spine by maintaining proper posture to prevent spinal changes and injury [1]. To maintain daily life performance without restriction, having normal spine range of motion (ROM) values is vital, ensuring that spinal movement remains within normal movement limits to provide sustained spinal stability [2]. Millions have relied on backpacks to answer daily requirements since the early decades, and their design has improved the comfort of their carriers and alleviated the physical strain of carrying substantial loads [3]. During the twentieth century, concerns regarding the potential health consequences of backpack usage began to surface [4]. Many studies were conducted in response to a growing awareness of the possible effects on users, especially those carrying heavy loads for prolonged periods, to explore the effects of carrying heavy backpacks on back muscle activity and the range of motion (ROM) of the joints, considering a heavy backpack might change them [5]. While some researchers tried to find alternative backpack designs to mitigate possible risks [6], for adolescents, some other researchers, including Pang et al., studied if trolley bags would be an alternative to backpacks when walking on flat surfaces [7].

Carrying a heavy backpack can significantly change the force and strain amplitude on several back muscles, including the trapezius, Latissimus dorsi, Erector spinae, and Multifidus, which may stress joints and potentially cause spinal instability and poor posture [8–11]. Furthermore, a heavy backpack may reduce the ROM of the spine, change the joint angles, and alter the body posture and alignment [4]. Altering body posture and alignment may increase the risk of injury or discomfort [11]. Orozco et al. suggest that an increase in the inclination leads to changes in biomechanical and neuromuscular responses during uphill walking, specifically lower extremity muscle activation [12]; Cao et al. used 8% inclination (approximately 4.57$^{\circ }$) as the upper limit of low-slope urban area [13].

This study aims to understand how back muscle activations, joints’ range of motion (ROM), and their correlations change with the introduction of a backpack load while walking on a flat surface and an inclined surface. Understanding these changes is essential for clinicians and possibly backpack manufacturers to develop strategies or products to mitigate the potential adverse effects of backpacks so that they can ensure the safety and well-being of individuals, especially young adults who frequently carry them.

The joints explored in this study are the spinal motion segments (T1-C7, T8-T9, LI-T12, L4-L3, L5-S1) and lower extremities (hip, knee, and ankle joints). To our knowledge, this is the first study to examine change in muscle activities and joint movements in a single study in young adult subjects walking with a backpack on both level and inclined surfaces, an important topic in biomechanics.

## Methodology

### Participants

Twenty adults (15 males and five females) with an average age of 24.2 (19–28) participated in this study; the average height and weight were 174.2 (155–188) cm and 74.8 (55–102) kg, respectively.

Individuals with no cognitive impairment or scoliosis, no surgery or orthopedic history related to the spine, pelvis, legs, or feet, and no known joint movement limitations were accepted as subjects in this study. All subjects were right-handed and presumed to be right-footed [14]. The study obtained ethical approval from the ethical committee of Bahcesehir University, Istanbul, and was conducted at the Biomechanics Laboratory of Bahcesehir University. It complies with the institutional and national research committee’s ethical standards, the 1964 Helsinki Declaration, and its later amendments or comparable ethical standards.

### Backpack

In experiments, 30 $\times$ 42 $\times$ 11 cm backpacks were used, with a typical design of two straps between the top and the lower side.

After a preliminary survey with 28 participants, in line with their typical weight, backpack weight was adjusted to be 12% of the participating subject’s body weight.

### Data collection

For electromyography (EMG), the Trigno® Wireless Biofeedback (SN: SP-W02C-1357) System was used to record the EMG signals [15], according to SENIAM.org standards [16].

Joint angles and variations in the range of motions (ROM) were measured in degrees using XSENS MVN motion capture system [17]. Later, raw data were converted into a 23-segment avatar model, according to International Society of Biomechanics (ISB) standards, using 22 joint angle data using the MVN Analyze software [18].

### Experiment protocol

For baseline measurements, subjects first walked on a treadmill (4 km/h) at least 200 steps on flat (0$^{\circ }$) and on inclined (5$^{\circ }$) surfaces. After resting for 7 min, subjects walked with a backpack with the same speed and slope. A 5$^{\circ }$ slope is selected to represent the upper slope limit of low-slope urban area [13].

### Data conditioning and statistical analysis

EMG and motion data were synchronized using the Delsys Trigger Module. Raw surface EMG data was first detrended and later conditioned with a fourth-order 10 Hz low pass Butterworth filter. Calculated muscle activation root mean square (RMS) values were used to characterize the muscle activity.

Range of motion (ROM) of joints is calculated as the average of the differences of the maximum and the minimum displacements of joints during gaits [19].

D’Agostino-Pearson test was used as the normality test. Student’s paired *t*-test was used to analyze the effect of the backpack on EMG RMS voltages and ROMs. Pearson’s correlation coefficients between EMG and ROM signals were also calculated and scaled using Dancey and Reidy’s scale [20]. Unless otherwise specified, the statistical significance level was kept as ($p<0.05$) in all tests.

**Table 1:** Percent change in average muscle RMS activation levels

	Flat		Inclined$^{1}$	
	Left	Right	Left	Right
Erector spinae	$-$34.9	$-$33.4	$-$23.6	$-$18.3
Multifidus	$-$42.3	$-$16.8	$-$22.1	$-$20.9

$$^{1}$$Slope is 5$$^{\circ }$$

**Table 2:** Percent change in average joint range of motions$^{1}$

	Flat			Inclined		
ROM$^{2}$	LB	AB	FE	LB	AB	FE
Spine						
L5-S1	$-$35.2	$-$68.3	NS	$-$35.8	$-$67.3	NS
L4-L3	$-$33.5	$-$69.3	NS	$-$32.6	$-$67.4	NS
L1-T12	$-$33.5	$-$69.3	NS	$-$32.7	$-$67.4	NS
T9-T8	$-$35.1	$-$69.4	NS	$-$35.4	$-$67.3	NS
T1-C7	$-$13.3	$-$17.3	NS	NS	$-$19.6	NS
	AA	IE	FE/DP	AA	IE	FE/DP
R. lower body						
Hip	$-$12.1	22.4	6.3	$-$5.7	24.5	9.7
Knee	NS	NS	$-$4.2	NS	NS	NS
Ankle	6.2	NS	NS	8.6	7.8	4.7
L. lower body						
Hip	$-$15	35.6	5.9	NS	26.4	6.6
Knee	NS	NS	$-$3.5	NS	NS	NS
Ankle	NS	NS	NS	8.4	NS	NS

$^{1}$NS: Change is statistically insignificant (p>0.05)

$^{2}$*LB* lateral bending, *AB* axial bending, *FE* flexion/extension, *AA* abduction/adduction, *IE* internal-external rotation, *DP* dorsiflexion/plantarflexion

## Results

**Fig. 1:**
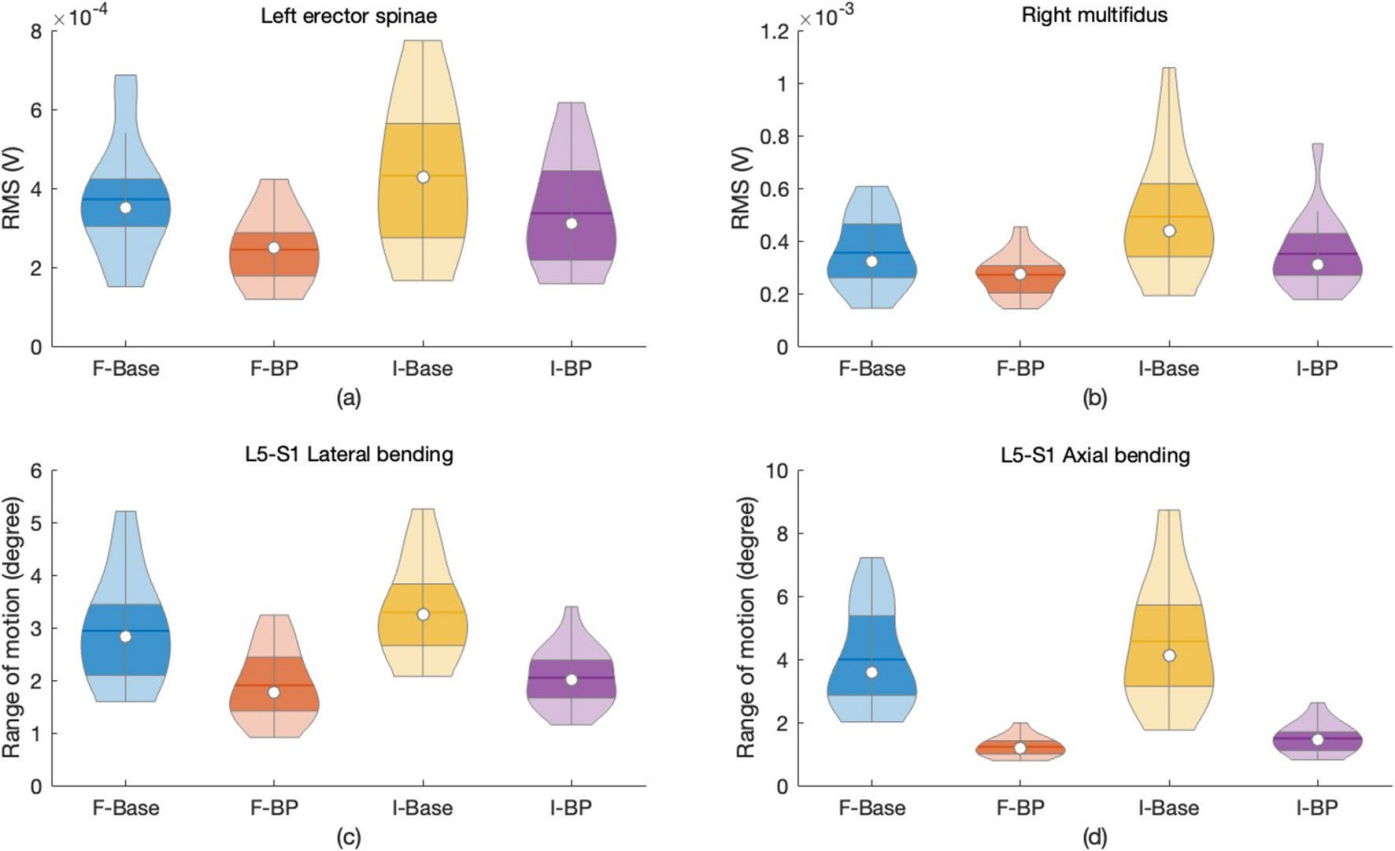
This shows how backpack load changes muscle activities and posture. After using the D’Agostino-Pearson test for their normality, Student’s paired *t*-test is used to understand if the effects of carrying a backpack were statistically significant compared to their baseline measurements. Both for flat and inclined surface walk scenarios, the back muscle activities were significantly decreased ($p<0.05$): **a** for left erector spinae activity, the reductions were 34.9% and 33.4%, respectively, and **b** for the right multifidus, the reductions were 16.8% and 20.9%, respectively. Compared to their baseline measurements, reductions in the range of motion of spinal joints (ROM) with backpack load were also statistically significant ($p<0.05$): **c** for flat and inclined surface walk scenarios, the reductions in lateral bending ROM of the L5-S1 joint were 35.2% and 35.8%, respectively, and **d** for its axial bending, the reductions were 69.3% and 67.3%. Different walk scenarios are coded as F-Base, flat surface baseline; F-BP, flat surface with backpack load; I-Base, inclined surface baseline; and I-BP, inclined surface with backpack load

Carrying a backpack significantly decreased EMG RMS voltages of the left multifidus (LM), right multifidus (RM), left erector spinae (LE), and right erector spinae (RE) muscles during walking on both flat and inclined surfaces ($p < 0.05$). The change in erector spinae muscles was not statistically significant ($p > 0.05$). Detailed data are presented in Table 1. In flat surface walking, backpack loads significantly reduced the ROM of spinal joints in lateral and axial bending ($p < 0.05$), with reductions for L5-S1, L4-L3, L1-T12, and T9-T8 joints ranging from 68.3 to 69.4% for lateral bending and 33.5 to 35.2% for axial bending. During inclined walking, reductions were similar: 67.3 to 67.4% for lateral bending and 32.6 to 35.8% for axial bending. Both in the flat and inclined surface walk, the T1-C7 joint showed the smallest decrease in axial bending (17.3–19.6%) and lateral bending (13.3%). Changes in spinal joint ROM during flexion-extension were not significant ($p > 0.05$). These results are summarized in Table 2. The ROM of lower body joints was also affected. Hip joints exhibited significant increases in internal-external rotation ROM ($p < 0.05$), while no significant changes were observed for knee or ankle ROMs. Details are presented in Table 2. Figure 1 displays how muscle activations and joint ROMs change by the backpack. Correlation analysis revealed significant associations between back muscle activation levels and joint ROMs during backpacked walking on flat surfaces. The strongest positive correlation was between the left erector spinae muscle and lateral bending of the L5-S1 joint ($r = 0.723$, $p < 0.001$), while the strongest negative correlation was between the right multifidus muscle and axial bending of the L5-S1 joint ($r = -0.577$, $p < 0.001$). Ankle joints showed moderate correlations with back muscle activation, but knee joint correlations were not statistically significant. These correlations are summarized in Table 3. During inclined surface walking, the left erector spinae muscle showed moderate positive correlations with lateral and axial bending of selective spinal joints, with the highest correlation for L5-S1 lateral bending ($r = 0.591$, $p = 0.0086$). Negative moderate correlations were observed between the left erector spinae muscle and ankle dorsiflexion-plantarflexion ($p < 0.05$). Correlations are summarized in Table 3.

**Table 3:** Correlation coefficients *r* between the joint motion and back muscle RMS voltages, while subjects were carrying a backpack$^{1}$

	Without slope				With slope
Joint movement$^{2}$	LE	RE	LM	RM	LE
*N=20*					
T8-T9					
AB	NS	NS	NS	$-$0.58	0.58
FE	NS	NS	0.70	0.48	NS
LB	0.72	NS	NS	NS	0.59
T12-L1					
AB	NS	$-$0.60	NS	$-$0.53	0.55
FE	NS	NS	0.70	0.48	NS
LB	0.72	NS	NS	NS	0.59
L3-L4					
AB	NS	$-$0.59	NS	$-$0.53	0.55
FE	NS	NS	0.70	0.48	NS
LB	0.72	NS	NS	NS	0.59
L5-S1					
AB	NS	NS	NS	$-$0.58	0.58
FE	NS	NS	0.70	0.48	NS
LB	0.72	NS	NS	NS	0.60
L. Hip					
IE	$-$0.57	$-$0.48	NS	$-$0.58	NS
R. Ankle					
IE	NS	NS	0.54	NS	$-$0.50
L. Ankle					
IE	NS	NS	0.48	NS	$-$0.67

$^{1}$NS: Correlation between the joint rotation and the back muscle RMS voltages is statistically not significant ($p>0.05$)

$^{2}$ *LE* left erector spinae, *LM* left multifidus, *RE* right erector spinae, *RM* right multifidus, *AB* axial bending, *DP* dorsiflexion planterflexion, *FE* flexion/extension, *LB* lateral bending, *IE* internal-external rotation

## Discussion

The study shows that carrying a backpack may significantly change joint ROM and back muscle activation levels while walking.

The reduction in multifidus muscle activities, specifically lateral and axial bending, suggests postural adjustments to accommodate the load. These adjustments may involve redistributing muscle effort to maintain balance and minimize strain on specific muscle groups, possibly explaining the decrease in erector spinae activation. This finding also supports the work of Jamshidnejad et al., who indicated that the body might develop a strategy to align its muscle activities based on changing demands [21].

The observed reductions in spinal joint ROM, especially in the lumbar region (e.g., L5-S1 and L4-L3), indicate restricted flexibility and increased stiffness, potentially due to load-induced stress and compression of intervertebral discs. Such stress may lead to long-term consequences, including back pain or discomfort if backpacks are carried frequently or improperly; this is in line with the findings of Suri et al. who suggest that high backpack loads alter active and passive responses of lower back tissues and may contribute to discomfort and long-term back pain [22].

Interestingly, the relatively small changes in cervical joint ROM (e.g., T1-C7) suggest that the upper spine may adapt better to load-induced demands, possibly due to its role in stabilizing the head, which is also supporting the findings of Dave et al. who studied the compensatory mechanisms of cervical region for head stability during dynamic activities [23].

The increased ROM of hip joints in internal-external rotation reflects a biomechanical response to compensate for restricted spinal movement, and Pang et al. suggest heavy backpack load causes forward leaning of trunk [7]. The adaptation in the hip may be for maintaining balance and forward propulsion during walking and highlighting the interconnectedness of the spine and lower limb biomechanics. This finding supports the previous search of Liu et al. in which the effect of load on hip and limb muscles was extensively investigated [24].

Correlation analysis underscores the asymmetric and load-dependent relationship between back muscle activity and joint ROMs. For example, the strong positive correlation between left erector spinae activity and the L5-S1 ROM suggests that this muscle plays a critical role in stabilizing the lower back under load. The lack of correlation at baseline measurements suggests that backpacks may significantly alter the biomechanical interplay between muscles and joints which is also consistent with the findings of Li et al. [25]. Furthermore, the asymmetry of the correlations between the muscles and the ROM of the joints suggests that subjects have adjusted their muscle activities, posture, and gait patterns asymmetrically and probably distributed load on their preferred legs unevenly to handle the external load better.

By understanding that heavy backpacks significantly alter joint angles, their range of motions, and the activation of the muscles that support those joints, clinicians can develop some targeted interventions to mitigate the short and long-term adverse effects of backpacks. Clinicians may recommend proper backpack usage to individuals and prescribe exercises to strengthen the most affected muscles, and they may work with manufacturers in more ergonomic backpack designs. Finally, with this understanding, clinicians will be able to address issues early so that young adults can maintain a good posture, overall musculoskeletal health, and an enhanced quality of life with a reduced risk of injury. However, the study has also some limitations. First, fixed treadmill speed may not align with individuals’ preferred gait patterns, restricting natural variations in gait cycles and creating inconsistent exercise difficulty. Second, the backpack was of a single size, with participants adjusting the strap length for comfort; hence, the variability introduced by the backpack position in the results is unknown. Third, the study sample constituted of young adults of mixed genders, limiting the generalizability of the findings to other age groups and gender. Fourth, only a 5$^{\circ }$ slope was used except for flat surface measurements, and how the musculoskeletal responses were changing with varying slope levels remains to be determined. Finally, the study sample size was relatively small. Therefore, future research with larger participant cohorts exploring the effects of these variables may help refine this study’s findings.

## Conclusion

To our knowledge, this study is the first attempt to simultaneously include EMG and biomechanical analysis in a single work to understand the effects of carrying a backpack in young adults, both for level and inclined surface walking. The study provided valuable insights into the affected muscles, the limitations on joint movements, and the asymmetric correlations between them.
